# PGK1 Is a Key Target for Anti-Glycolytic Therapy of Ovarian Cancer: Based on the Comprehensive Analysis of Glycolysis-Related Genes

**DOI:** 10.3389/fonc.2021.682461

**Published:** 2021-07-01

**Authors:** Rui Gou, Yuexin Hu, Ouxuan Liu, Hui Dong, Lingling Gao, Shuang Wang, Mingjun Zheng, Xiao Li, Bei Lin

**Affiliations:** ^1^ Department of Obstetrics and Gynaecology, Shengjing Hospital Affiliated to China Medical University, Shenyang, China; ^2^ Key Laboratory of Maternal-Fetal Medicine of Liaoning Province, Key Laboratory of Obstetrics and Gynecology of Higher Education of Liaoning Province, Liaoning, China; ^3^ Department of Obstetrics and Gynecology, University Hospital, LMU Munich, Munich, Germany

**Keywords:** PGK1, Warburg effect, ovarian cancer, anti-glycolytic therapy, NG52

## Abstract

Reprogramming of energy metabolism is a key hallmark of cancer, which provides a new research perspective for exploring the development of cancer. However, the most critical target of anti-glycolytic therapy for ovarian cancer remains unclear. Therefore, in the present study, Oncomine, GEPIA, and HPA databases, combined with clinical specimens of different histological types of ovarian cancer were used to comprehensively evaluate the expression levels of glycolysis-related metabolite transporters and enzymes in ovarian cancer. We selected phosphoglycerate kinase 1 (*PGK1*), which showed the greatest prognostic value in the Kaplan-Meier Plotter database, for subsequent validation. Immunochemistry assays confirmed that PGK1 was highly expressed in ovarian cancer. The PGK1 expression level was an independent risk factor for the survival and prognosis of patients with ovarian cancer. Functional analysis showed that the *PGK1* expression level was positively correlated with the infiltration of neutrophils. Cell experiments confirmed that inhibiting PGK1 expression in ovarian cancer cells could reduce the epithelial-mesenchymal transition (EMT) process, resulting in loss of cell migration and invasion ability. The small molecule NG52 dose-dependently inhibited the proliferation of ovarian cancer cells. In addition, NG52 reduced the EMT process and reversed the Warburg effect by inhibiting PGK1 activity. Therefore, PGK1 is an attractive molecular target for anti-glycolytic therapy of ovarian cancer.

## Introduction

Ovarian cancer is a common malignant tumor of the female reproductive system (1). Due to its insidious onset and a lack of effective early diagnostic methods, approximately 75% of patients present with advanced stage (2). Currently, the main treatment for ovarian cancer is cytoreductive surgery and platinum-based combination chemotherapy (3). In addition, new targeted drugs such as Poly (ADP-ribose) polymerase (PARP) inhibitors have broad application prospects (4, 5). However, due to the limited population of the intended drug for application and chemotherapy resistance, some patients still fail to achieve desirable progression-free survival (PFS) (6). Therefore, clarifying the mechanism of ovarian cancer tumorigenesis and exploring potential therapeutic targets are essential to improve the prognosis of patients with ovarian cancer.

Reprogramming of energy metabolism is a key hallmark of cancer. Tumor cells adjust their metabolism and nutrition methods, thereby maintaining the ability to continually proliferate (7). Under aerobic conditions, normal cells produce ATP through the tricarboxylic acid (TCA) cycle and oxidative phosphorylation (OXPHOS), however, an increase in the rate of glycolysis is observed in tumor cells, termed the “Warburg effect”. Tumor cells take up a large amount of glucose through glucose transporters and produce pyruvate through 10 enzymatic reactions. Pyruvate is converted into lactic acid under the catalysis of lactate dehydrogenase, which is exported from the cell to form an acidic microenvironment and promote invasion, metastasis, and immune escape of tumor cells (8). Blocking aerobic glycolysis of tumor cells can reduce the energy supply and production of metabolic intermediates, thereby effectively inhibiting tumor progression (9). Therefore, targeting aerobic glycolysis has become the focus of anticancer drug research. However, limited data are available regarding altered expression level of genes involved in glycolysis in ovarian cancer. In addition, the existing anti-glycolytic therapy has not yet been translated into clinical practice and the most effective therapeutic target need to be explored.

Therefore, in the present study, bioinformatics analysis and experimental verification were combined to systematically analyze the expression levels of metabolite transporters and enzymes involved in glycolysis in ovarian cancer. A large number of clinical specimens were used to screen out phosphoglycerate kinase 1 (PGK1), which showed the strongest correlation with the prognosis of ovarian cancer patients. As the first ATP-producing enzyme in the glycolysis process, PGK1 plays a key role in coordinating energy production with biosynthesis and cellular redox (10). PGK1 is upregulated in numerous types of tumors, which is correlated with malignant biological behaviors of cancer cells (11). In the present study, we explored the role of PGK1 in epithelial-mesenchymal transition (EMT) process and immune evasion in ovarian cancer. In addition, we evaluated the significance of PGK1 as an attractive molecular target in the anti-glycolytic treatment of ovarian cancer and proposed the therapeutic effect of NG52 as a PGK1 inhibitor.

## Materials and Methods

### Data Extraction From Oncomine, GEPIA, and HPA Databases

The Oncomine database (http://www.oncomine.org), which is an online microarray database, was used to analyze the mRNA expression levels of glycolysis-related genes in different cancers (12). The following thresholds were set: gene grade = 10%; fold change > 2; *P* < 0.01.

Gene Expression Profling Interactive Analysis (GEPIA) (http://gepia.cancer-pku.cn/) is a database of RNA sequencing expression data retrieved from The Cancer Genome Atlas (TCGA) and Genotype-Tissue Expression (GTEx) projects (13). GEPIA was used to confirm the differential gene expression levels of glycolysis-related genes in ovarian cancer and adjacent tissues.

The Human Protein Atlas (HPA) database (https://www.proteinatlas.org) is dedicated to providing tissue and cell distribution information of all 24,000 human proteins (14). Each protein is detected in 64 cell lines, 48 normal human tissues, and 20 tumor tissues using immunoassay technology. The protein expression of glycolysis-related genes in ovarian cancer was validated using immunohistochemical assays.

### Kaplan-Meier Plotter Analysis

As a biomarker evaluation tool, Kaplan-Meier Plotter (http://kmplot.com) can be used to evaluate gene expression data and survival information of patients with ovarian cancer (15). The prognostic value of glycolysis-related genes was assessed using a Kaplan-Meier survival plot, with the hazard ratio (HR), 95% confidence interval (CI), and log-rank *P*-value.

### Functional and Pathway Enrichment Analysis

As an open database based on the TCGA database, the cBioPortal database (www.cbioportal.org) includes various data such as DNA copy, mRNA, and microRNA expression (16). The database was used to analyze the expression correlation of *PGK1* and other genes. A Spearman’s correlation coefficient exceeding 0.40 indicated a good correlation.

Metascape (http://metascape.org) integrates multiple authoritative databases, such as Gene Ontology (GO), Kyoto Encyclopedia of Genes and Genomes (KEGG), UniProt, and DrugBank, to provide gene enrichment, biological process annotation, and protein interaction network analysis (17). In the present study, Metascape was used to evaluate the GO and KEGG enrichment of *PGK1* and its co-expressed genes.

### Immune Infiltration Analysis

TISIDB (http://cis.hku.hk/TISIDB) is an online analysis website that contains a variety of immunological data that can be used to analyze the interaction between tumors and the immune system (18). We analyzed the correlation between *PGK1* and tumor-infiltrating cells using the TISIDB database. In addition, the TIMER database (http://timer.cistrome.org) was used to confirm the correlation (19).

### Sample Sources and Clinical Data

Paraffin specimens of ovarian tissue were obtained from surgically removed ovarian tissues of inpatients in the Shengjing Hospital of China Medical University. One specimen each of ovarian serous carcinoma, mucinous carcinoma, endometrioid carcinoma, clear cell carcinoma, and normal tissue was selected for serial sectioning to detect the protein levels of glycolysis-related genes. In addition, a total of 140 paraffin specimens of ovarian tissues surgically removed from hospitalized patients from 2008 to 2012 were selected to evaluate the expression level and prognostic value of PGK1. Pathological sections were diagnosed by pathologists and divided into the following four groups: epithelial ovarian cancer (malignant tumor group, n = 103); epithelial ovarian borderline tumors (borderline tumor group, n = 14); epithelial ovarian benign tumors (benign tumor group, n = 13); normal ovarian tissue (normal ovary group, n = 10). The median age in the four groups was 54 years (13–84 years of age) and significant difference in age was not observed between the four groups (*P* > 0.05). Patients did not undergo radiotherapy, chemotherapy, or hormone therapy before surgery. This study was approved by the Ethics Committee of China Medical University and written informed consent was obtained from all participants.

### Immunohistochemistry

Paraffin specimens of ovarian tissue were processed into 5-μm-thick sections. The streptavidin-peroxidase ligation (SP) method was used to detect the protein expression of glycolysis-related genes. Control experiments were set for each batch. The primary antibodies are shown in **Additional File 1**. The immunohistochemical staining results were considered positive when brown particles were observed in the cell membrane and cytoplasm. The coloring intensity was divided into not pigmented, light yellow, brownish-yellow, and dark brown, which was recorded as 0, 1, 2, and 3 points, respectively. The percentage of stained cells within the microscope field of view was classified into < 5%, 5–25%, 26–50%, 51–75%, and > 75%, which were recorded as 0, 1, 2, 3, and 4 points, respectively. The final score was obtained by multiplying the two scores and categorized as follows: 0–2 points (–), 3–4 points (+), 5–8 points (++), and 9–12 points (+++). Two observers assessed each tissue section independently to reduce errors.

### Cell Culture

Ovarian cancer cell lines (OVCAR3, A2780, and ES-2) were purchased from the Shanghai Cell Collection Center. OVCAR3 and A2780 cells (adenocarcinoma) were cultured with RPMI 1640 medium containing 10% fetal bovine serum, and the ES-2 cell (clear cell carcinoma) was cultured with McCoy’s 5A medium containing 10% fetal bovine serum. Cells were cultured in a 37°C incubator with 5% CO_2_ and saturated humidity. The liposome method (Lipo 3000 transfection kit, GIBCO, Invitrogen, Carlsbad, CA, USA) was used to transfect the PGK1 small interfering (si) RNA into OVCAR3 and ES-2 cell lines. *PGK1* siRNA#1 (GenePharma, Shanghai, China) sequences were as follows: sense: 5’-CCAAGUCGGUAGUCCUUAUTT-3’; antisense: 5’-AUAAGGACUACCGACUUGGTT-3’. *PGK1* siRNA#2 sequences were as follows: sense: 5’-GCUUCUGGGAACAAGGUUATT-3’; antisense: 5’-UAACCUUGUUCCCAGAAGCTT-5’. Control siRNA sequences were as follows: sense: 5’-UUCUCCGAACGUGUCACGUTT-3’; antisense: 5’-ACGUGACACGUUCGGAGAATT-3’. After 48 hours of transfection, cells were collected to detect the interference effect using western blotting.

### Western Blot

Cells were collected and lysed with pre-cooled RIPA lysis buffer and the protein concentration was quantified using the BCA method. The protein was separated on 10% SDS-PAGE gel and transferred to polyvinylidene difluoride (PVDF) membrane (Millipore, Billerica, MA, USA). The membrane was blocked with 5% milk for 2 hours. Next, the membrane was incubated with the following primary antibody at 4°C overnight: anti-PGK1 (1:1000; 17811-1-AP; Proteintech, Wuhan, China); anti-MMP2 (1:1000; 10373-2-AP; Proteintech); anti-MMP9 (1:1000; 10375-2-AP; Proteintech); anti-E-cadherin (1:5000; 20874-1-AP; Proteintech); anti-N-cadherin (1:5000; 22018-1-AP; Proteintech). Tubulin was used as an internal control. The membrane was incubated with the secondary antibody (1:5000, Zhongshan Jinqiao, China) for 2 hours at room temperature and visualized using the ECL reagent (Thermo Fisher Scientific ECL, Carlsbad, CA, USA).

### CCK-8 Assays and Colony Formation Assays

Cells were seeded into 96-well plates at 2000 cells/well. After 6 hours of cell adhesion, time was recorded as 0 time point. Next, the CCK8 solution (Bimake, Houston, TX, USA) was added to cells at 0, 24, 48, 72, and 96 hours and incubated for 2 hours. Under the dosing conditions, cells were cultured for 12 hours, after which NG52 at the specified concentration was added. The optical density (OD) values were measured using a universal microplate reader at 450 nm.

Cells were seeded into 6-well plates at 500 cells/well and cultured for 10 days. The colonies were fixed with 4% paraformaldehyde for 15 minutes and stained with crystal violet. The colonies were photographed and counted.

### Scratch Assay and Invasion Assay

Exponentially growing cells were trypsinized and plated in 6-well plates. When the cell fusion reached 90%, the 6-well plates were scratched with a 100 µL pipette tip. Cells were cleared with PBS three times and then cultured in serum-free culture medium for 24 hours. The scratch width was observed under the microscope.

The invasion ability of ovarian cancer cells was assessed using a transwell chamber. The upper side of the chamber was uniformly coated with 70 µL Matrigel glue (BD Corporation, Franklin Lakes, NJ, USA). Then, 200 µL single cell suspension (2 × 10 ^5^ cells) diluted with serum-free medium was added to the upper chamber, and 500 µL medium containing 20% fetal bovine serum to the lower chamber. After 48 hours of incubation, cells were fixed with 4% paraformaldehyde for 30 minutes and stained with crystal violet for 30 minutes. Pictures were taken with a microscope and the number of stained cells counted.

### Apoptosis Assay

The apoptosis assay was performed using the Annexin-V-fluorescein isothiocyanate (FITC)/propidium iodide (PI) (KeyGen Biotech, Nanjing, China) staining method when cells were treated with different concentrations of NG52. Assays were performed according to the instructions of the manufacturer.

### Extracellular Acidification Rate (ECAR) and Oxygen Consumption Rate (OCR)

Seahorse Bioscience XF96 Extracellular Flux Analyzer (Seahorse Bioscience, Agilent Technologies, USA) was used to measure cellular glycolytic capacity and mitochondrial function, following the manufacturer’s instructions of Seahorse XF Glycolysis Stress Test Kit or Seahorse XF Cell Mito Stress Test Kit. Cells were plated in XF96 Cell Culture Microplates (6×10^3^ cells/well) and cultured overnight. Then, the cells were treated with NG52 for 12 hours prior to the measurement. For ECAR analysis, 10 mM glucose, 1 μM oligomycin, and 50 mM 2-deoxy-glucose were added automatically. For OCR analysis, 1.5 μM oligomycin, 1 μM FCCP, and 0.5 μM rotenone/antimycin A were added automatically. Seahorse XF-96 wave software was used to calculate the data.

### Activity Assay

Cells were plated in 6-well plates. After attachment, cells were treated with NG52 in different concentrations and cultured for 12 hours. Ammonium sulfate precipitation method was used to remove small molecules. The assay was performed using the phosphoglycerate kinase activity kit (ab252890, Abcam) to test the inhibitory effect of NG52 on PGK1 kinase activity. Absorbance of each sample at 340 nm was measured at 0 min and 60 min. The relative activity was calculated according to the absorbance values and NADH standard curve.

### Statistical Analysis

Data were analyzed using the SPSS 22.0 software (IBM Corporation, Armonk, NY, USA). The count data were analyzed using the chi-squared and Fisher’s exact probability tests, and the measurement data were analyzed using the Student’s *t*-test. Survival curves were analyzed using the Kaplan-Meier and log-rank tests. The Cox regression model was used to analyze the prognosis value of PGK1. GI_50_ value was calculated using Prism 7 (GraphPad Software, San Diego, CA, USA). *P* < 0.05 was considered statistically significant.

## Results

### Expression Level of Glycolysis-Related Genes

**Figure 1:**
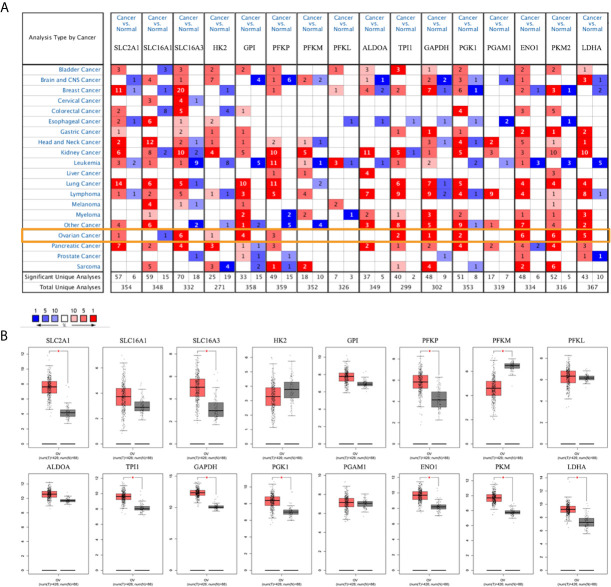
mRNA expression levels of glycolysis-related genes. **(A)** mRNA expression levels of glycolysis-related genes in various types of cancer (Oncomine). **(B)** mRNA expression levels of glycolysis-related genes in ovarian cancer and normal ovarian tissues (GEPIA). Note: *SLC2A1*, *SLC16A1*, *SLC16A3* are genes encoding GLUT1, MCT1, and MCT4. There is no *PKM2* expression data in the GEPIA database. *P < 0.01.

To evaluate the role of glycolysis in ovarian cancer progression, Oncomine and GEPIA databases were used to analyze the mRNA expression level of the metabolite transporters and enzymes involved in glycolysis in ovarian cancer. Except for the partial enzyme data that did not meet the screening criteria, most glycolysis-related genes were highly expressed in ovarian cancer, especially solute carrier family 16 member 3 (*SLC16A3*), enolase 1 (*ENO1*), and pyruvate kinase M2 (*PKM2*), which have been confirmed by six studies in the Oncomine database (**Figure 1A**). Notably, the solute carrier family 16 member 1 (*SLC16A1*) expression level in ovarian cancer was lower than in normal ovarian tissue in the Oncomine database. The results were basically similar to the GEPIA database results (**Figure 1B**).

**Figure 2:**
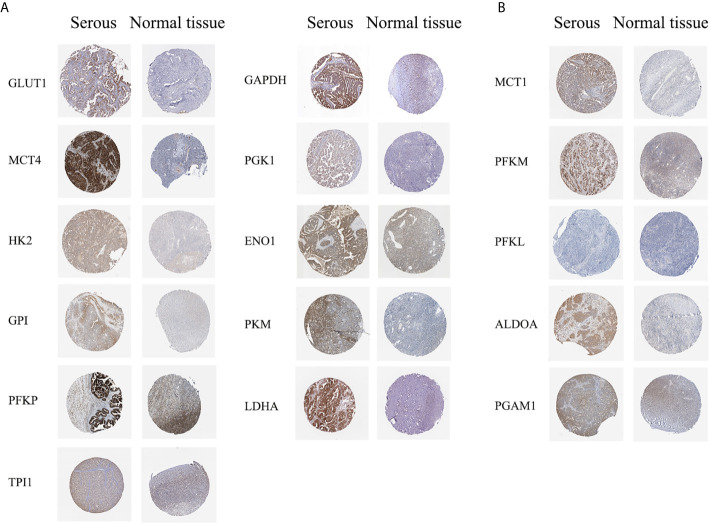
Protein expression levels of glycolysis-related genes (HPA dabatase). **(A)** Protein expression levels of high expression glycolysis-related genes. **(B)** Protein expression levels of other glycolysis-related genes.

The HPA database was used to further verify the protein expression levels of glycolysis-related genes in ovarian cancer tissues and normal tissues (**Figure 2**, **Additional file 2**). The expression levels of highly expressed indicators, which were confirmed in the Oncomine or GEPIA database, were higher in ovarian cancers than normal tissues. However, phosphofructokinase platelet (*PFKP*) and *ENO1* stained strong in normal tissues, which may be due to differences in antibody non-specificity. In addition, except for phosphofructokinase liver type (*PFKL*), the expression levels of other indicators that were not confirmed to have high expression in ovarian cancer were slightly darker in ovarian cancer tissues than in normal tissues.

**Figure 3:**
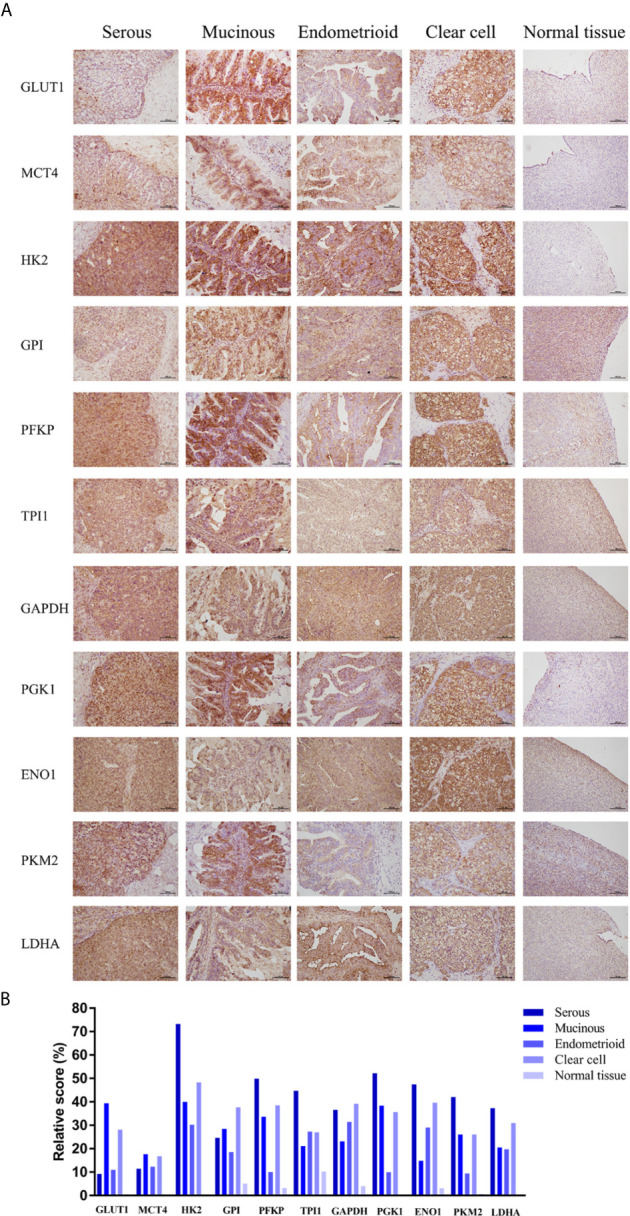
Expression levels of glycolysis-related proteins in clinical samples. **(A)** Representative immunohistochemistry images of glycolysis-related proteins in epithelial ovarian cancer and normal tissue. **(B)** Immunostaining analysis using ImageJ.

Based on the results, solute carrier family 2 member 1 (*SLC2A1*), *SLC16A3*, hexokinase 2 (*HK2*), glucose-6-phosphate isomerase (*GPI*), *PFKP*, triosephosphate isomerase 1 (*TPI1*), glyceraldehyde-3-phosphate dehydrogenase (*GAPDH*), *PGK1*, *ENO1*, *PKM2*, and lactate dehydrogenase A (*LDHA*), which showed high mRNA expression level in the Oncomine or GEPIA database, were selected for immunohistochemical assays. Serial sections of the same patient were taken to compare the protein expression differences of the above-listed indicators in different histological types of epithelial ovarian cancer and normal ovarian tissue (**Figures 3A, B**). The protein expression levels of the above-mentioned genes were more highly expressed in the ovarian cancer tissues than in the normal tissues. However, GPI, PFKP, TPI1, GAPDH, and ENO1 were slightly stained in the interstitial part of normal tissues.

### Prognostic Value of Glycolysis-Related Genes in Ovarian Cancer

**Figure 4:**
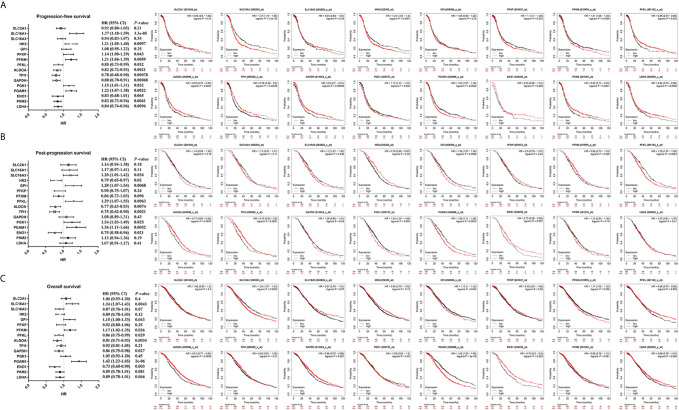
The prognostic value of glycolysis-related genes in ovarian cancer. **(A)** The prognostic value of glycolysis-related genes in ovarian cancer (PFS in Kaplan-Meier plotter). **(B)** The prognostic value of glycolysis-related genes in ovarian cancer (PPS in Kaplan-Meier plotter). **(C)** The prognostic value of glycolysis-related genes in ovarian cancer (OS in Kaplan-Meier plotter).

To further identify metabolite transporters and enzymes that showed the most clinical significance in the glycolysis process of ovarian cancer, the Kaplan-Meier Plotter database was used to analyze the correlation between the expression levels of glycolysis-related genes and the survival of patients with ovarian cancer (**Figure 4**). Among the highly expressed glycolysis-related genes, high *GPI* expression was associated with poor prognosis of post-progression survival (PPS) and overall survival (OS) in patients with ovarian cancer, and high expression of *PGK1* was associated with poor PFS and PPS. In addition, upregulation of *HK2* and *PFKP* showed significant correlations with poor PFS, and upregulation of *SLC16A3* showed significant correlations with poor PPS. Although database analyses showed no significant difference in phosphoglycerate mutase 1 (*PGAM1*) expression level in ovarian cancer and normal tissues, the patients with high *PGAM1* level were predicted to have poor PFS, PPS, and OS, which may be due to the inclusion of different ethnic and age groups in different databases.

**Figure 5:**
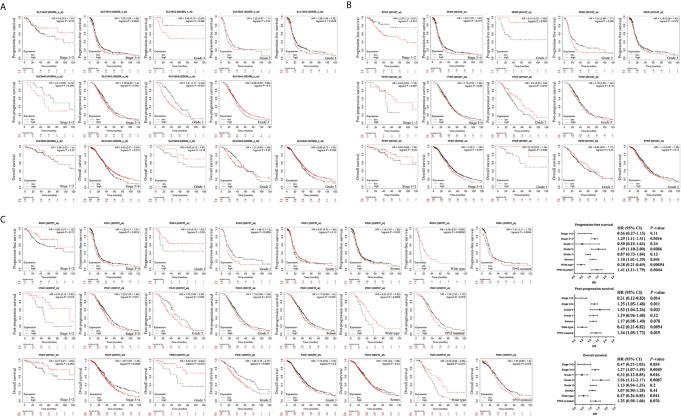
The prognostic value of *SLC16A3*, *PFKP*, and *PGK1* in ovarian cancer. **(A)** The correlation between *SLC16A3* expression level and the prognosis of PFS, PPS, and OS in ovarian cancer. **(B)** The correlation between *PFKP* expression level and the prognosis of PFS, PPS, and OS in ovarian cancer. **(C)** The correlation between *PGK1* expression level and the prognosis of PFS, PPS, and OS in ovarian cancer.

*SLC16A3*, *PFKP*, and *PGK1*, which were highly expressed in ovarian cancer tissues in two databases and associated with poor prognosis, were selected for further analysis (**Figure 5**). Results indicated that high *SLC16A3* and *PFKP* expression in patients with advanced ovarian cancer was associated with poor PFS, and high *SLC16A3* expression in patients with poor or moderate differentiation was associated with poor PFS or PPS (**Figures 5A, B**). For patients with International Federation of Obstetrics and Gynecology (FIGO) stage III–IV and moderate differentiation, upregulation of *PGK1* expression indicated poor PFS, PPS, and OS (**Figure 5C**). Therefore, *PGK1* showed the greatest prognostic value for patients with ovarian cancer.

Further results showed high *PGK1* expression in patients with serous ovarian cancer indicated a poor prognosis of PFS. In addition, a significant correlation was observed between *PGK1* mRNA upregulation and poor PFS and PPS in patients with *TP53* mutations compared with patients with wild-type *TP53*. Therefore, *PGK1* may play a key role in glycolysis of ovarian cancer and be used as a biomarker for evaluating poor prognosis in patients with ovarian cancer.

### Functional and Pathway Analyses of *PGK1* in Ovarian Cancer

**Figure 6:**
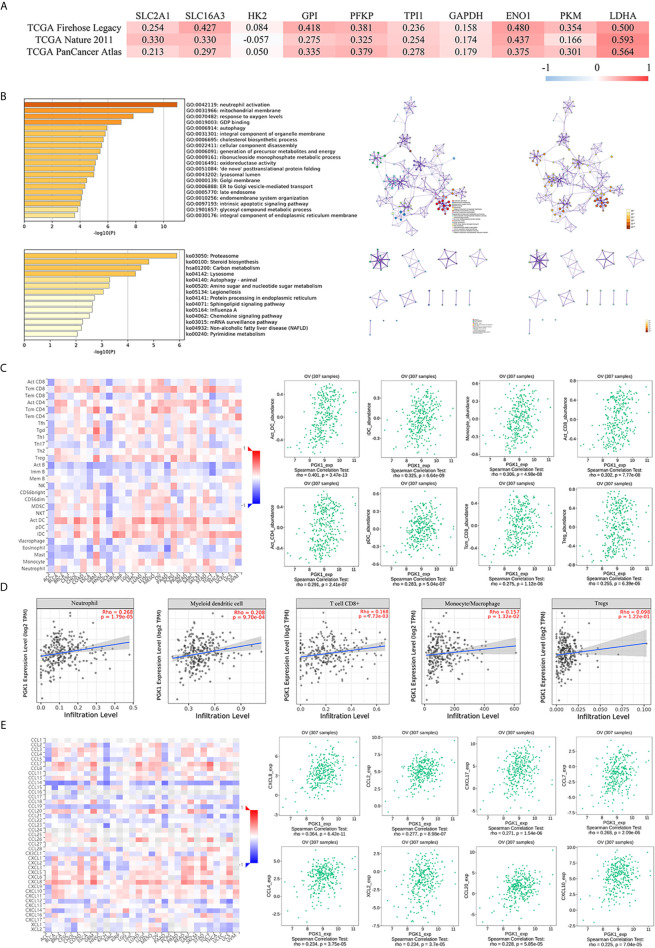
Functional and pathway analyses of *PGK1* in ovarian cancer. **(A)** The correlation between *PGK1* and other glycolysis-related genes. **(B)** GO and KEGG analyses of genes interacting with *PKG1*. **(C)** The correlation between *PGK1* and immune or inflammatory cell infiltration (TISIDB database). **(D)** The correlation between *PGK1* and immune or inflammatory cell infiltration (TIMER database). **(E)** The correlation between *PGK1* and chemokines (TISIDB database). There is no *PKM2* expression data in the cBioPortal database.

Based on the results showing the role of *PGK1* in glycolysis of ovarian cancer, the cBioPortal database was used to analyze the correlation between *PGK1* expression and other glycolysis-related genes in ovarian cancer. The results showed that highly expressed glycolysis-related genes, except for *HK2*, were positively correlated with *PGK1* (*P* < 0.05). *PGK1* had the best correlation with *LDHA*, and Spearman’s correlation coefficient between *SLC16A3*, *GPI*, *ENO1*, *LDHA*, and *PGK1* exceeded 0.40 in the TCGA Firehose Legacy data (**Figure 6A**); consistency in results among the three studies was good.

A total of 217 genes co-expressed with *PGK1* having an average Spearman’s correlation coefficient of 0.45 were obtained from the cBioPortal database based on the selection criteria. The genes co-expressed with *PGK1* were subjected to functional and pathway enrichment analyses using Metascape (**Figure 6B**). Among biological processes showing the greatest correlation for genes co-expressed with *PGK1*, GO: 0006695 (cholesterol biosynthetic process), GO: 0006091 (generation of precursor metabolites and energy), GO: 0009161 (ribonucleoside monophosphate metabolic process), GO: 0016491 (oxidoreductase activity), and GO: 1901657 (glycosyl compound metabolic process) were correlated with metabolic reprogramming. Surprisingly, *PGK1* and its co-expressed genes participated in neutrophil activation (GO: 0042119) and chemokine signaling pathway (ko04062). Neutrophils can become tumorigenic during aggressive stages of tumor progression. Inhibiting recruitment or activation can limit pro-tumorigenic capacity of neutrophils. Therefore, we further validated the correlation between *PGK1* and tumor-infiltrating cells.

The Spearman’s correlations between *PGK1* expression level and tumor-infiltrating cells were analyzed using the TISIDB database (**Figure 6C**). Cells displaying the greatest correlations included activated dendritic cell (Act_DC), interdigitating dendritic cell (iDC), monocyte, activated CD8+ T cell (Act_CD8), activated CD4+ T cell (Act_CD4), plasmacytoid dendritic cell (pDC), central memory CD8+ T cell (Tcm_CD8), and regulatory cell (Treg). The TIMER database confirmed that the infiltration levels of neutrophil, myeloid dendritic cell, CD8+ T cell, monocyte, macrophage, and Tregs were positively correlated with *PGK1* (**Figure 6D**). In addition, the correlation between *PGK1* and chemokines were analyzed to evaluate the role of *PGK1* in regulating neutrophil activation and recruitment. Chemokines displaying the greatest correlations included CXCL8 and CCL2 (**Figure 6E**).

### Validation of High PGK1 Expression in Epithelial Ovarian Cancer

**Figure 7:**
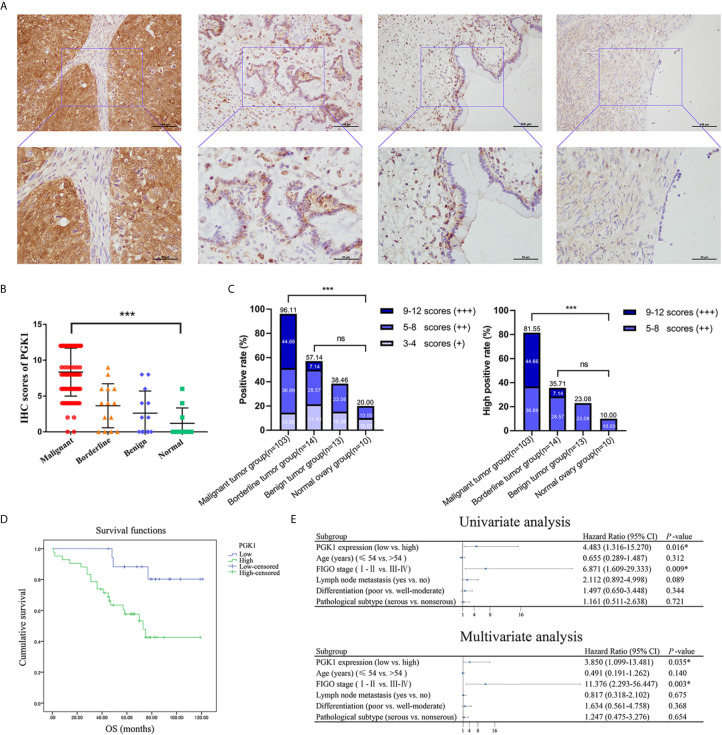
The PGK1 expression level in different groups of ovarian tissue and its prognostic value in epithelial ovarian cancer. **(A)** Representative immunohistochemical staining images of PGK1 in epithelial ovarian cancer, epithelial ovarian borderline tumor, epithelial ovarian benign tumor, and normal ovarian tissue. **(B)** PGK1 immunostaining scores in ovarian tissue. **(C)** Statistical analysis of PGK1 positive rate and high positive rate in different groups. **(D)** The correlation between PGK1 expression and OS in patients with epithelial ovarian cancer. **(E)** Forest plot based on univariate and multivariate analyses of patients with epithelial ovarian cancer. ***P < 0.001; ns, non-significant.

Immunohistochemistry assays showed that PGK1 was mainly localized on the cell membrane and in the cytoplasm (**Figure 7A**). Positive rate and high positive rate of PGK1 in the malignant tumor group (96.11% and 81.55%, respectively) were significantly higher than in the borderline tumor group (57.14% and 35.71%, respectively), benign tumor group (38.46% and 23.08%, respectively), and normal ovary group (20.00% and 10.00%, respectively). Positive rate and high positive rate between the borderline tumor group, benign tumor group, and normal ovary group were not significantly pairwise different (*P* > 0.05; **Figures 7B, C**).

### PGK1 Expression in Epithelial Ovarian Cancer Was Associated With Degree of Differentiation

A total of 103 patients with epithelial ovarian cancer were divided into high PGK1 expression group (++/+++) and low PGK1 expression group (-/+). Statistical analyses showed a significant correlation between PGK1 expression level and degree of differentiation (*P* = 0.015); patients with poor differentiation had significantly higher PGK1 high positive rate (90.57%) than patients with well or moderate differentiation (72.00%). PGK1 showed the highest high positive rate in mucinous carcinoma (100.00%) and the lowest high positive rate in endometrioid carcinoma (68.75%). However, the PGK1 expression level did not significantly correlate with FIGO stage, lymph node metastasis, or pathologic type (*P* > 0.05; **Table 1**).

**Table 1:** Relationships between PGK1 expression in epithelial ovarian cancer and clinicopathological parameters.

Characteristics	n	Low		High		High positive rate (%)	*P*-value
		(-)	(+)	(++)	(+++)		
**FIGO stage**							
I-II	39	1	7	11	20	79.49	0.673
III-IV	64	3	8	27	26	82.81	
**Differentiation**							
Well –moderate	50	3	11	14	22	72.00	0.015*
Poor	53	1	4	24	24	90.57	
**LN metastasis**							
No	75	3	13	24	35	78.67	0.216
Yes	28	1	2	14	11	89.29	
**Pathologic type**							
Serous	48	4	7	22	15	77.08	
Mucinous	10	0	0	2	8	100.00	0.246
Endometrioid	16	0	5	4	7	68.75	
Clear cell	11	0	1	3	7	90.91	
Poorly differentiated adenocarcinoma	18	0	2	7	9	88.89	

*P < 0.05.

### High PGK1 Expression Was an Independent Risk Factor Affecting the Survival and Prognosis of Epithelial Ovarian Cancer Patients

The 103 patients with epithelial ovarian cancer were followed up until April 30, 2019. Kaplan-Meier survival analysis showed the overall survival of patients in the high PGK1 expression group was shorter than that of patients in the low PGK1 expression group (*P* = 0.009, **Figure 7D**). The Cox regression model was used to analyze relationships between different clinicopathological parameters and prognosis. Univariate analysis indicated a significant correlation between PGK1 expression levels (*P* = 0.016), FIGO stages (*P* = 0.009), and the OS of patients. Multivariate analysis indicated PGK1 expression level (*P* = 0.035) and FIGO stages (*P* = 0.003) were independent risk factors affecting the survival and prognosis of patients with epithelial ovarian cancer (**Figure 7E**).

### Knockdown of PGK1 Attenuated EMT Process and Suppressed Glycolysis

**Figure 8:**
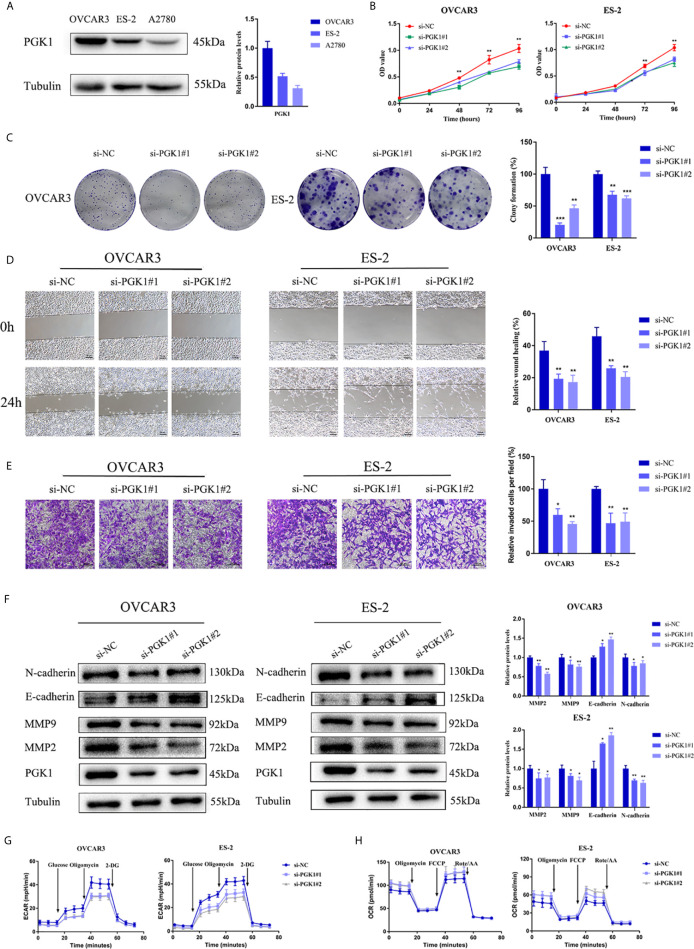
PGK1 affected EMT process and glycolysis of ovarian cancer cells. **(A)** PGK1 protein expression in three kinds of ovarian cancer cell lines. **(B)** PGK1-siRNA inhibited the proliferation of ovarian cancer cells. **(C)** PGK1-siRNA reduced the formation of colonies of ovarian cancer cells. **(D)** PGK1-siRNA reduced the migration of ovarian cancer cells. **(E)** PGK1-siRNA reduced the invasion of ovarian cancer cells. **(F)** PGK1-siRNA decreased the protein expression levels of MMP2, MMP9, and N-cadherin but increased the protein expression level of E-cadherin. **(G)** Chart of ECAR measurement in PGK1-silenced cells. **(H)** Chart of OCR measurement in PGK1-silenced cells. Error bars represent the mean ± SD of three independent experiments. *P < 0.05; **P < 0.01; ***P < 0.001.

To explore the function of PGK1 in ovarian cancer, its expression in cell lines was detected. The results showed that the expression of PGK1 was higher in the OVCAR3 and ES-2 cell lines than that in the A2780 cell line (**Figure 8A**). Therefore, OVCAR3 and ES-2 cell lines were transfected with siRNA, and the knockdown effect at the protein level was verified using western blot analysis. CCK-8 results confirmed that knockdown of PGK1 can inhibit cell viability, especially at 72 hours and 96 hours (**Figure 8B**). Similarly, when PGK1 was knocked down, the formed colonies of ovarian cancer cells were markedly reduced (**Figure 8C**). Compared with the control group, knockdown of PGK1 significantly reduced the migration and invasion ability of ovarian cancer (**Figures 8D, E**). To clarify the relevant mechanism, the expression level of EMT-related proteins was further investigated. Results showed that the expression levels of matrix metalloproteinases 2 (MMP2), MMP9, and N-cadherin decreased but those of E-cadherin increased after PGK1 inhibition (**Figure 8F**). By using Seahorse XF Extracellular Flux Analyzers, we examined the impact of PGK1 silencing on cellular glycolytic capacity and mitochondrial function. Knockdown of PGK1 in ovarian cancer cells decreased ECAR and increased OCR, reflecting the positive role of PGK1 in the regulation of aerobic glycolysis (**Figures 8G, H**).

### NG52 Induced Apoptosis and Attenuated EMT Process of Ovarian Cancer Cells

**Figure 9:**
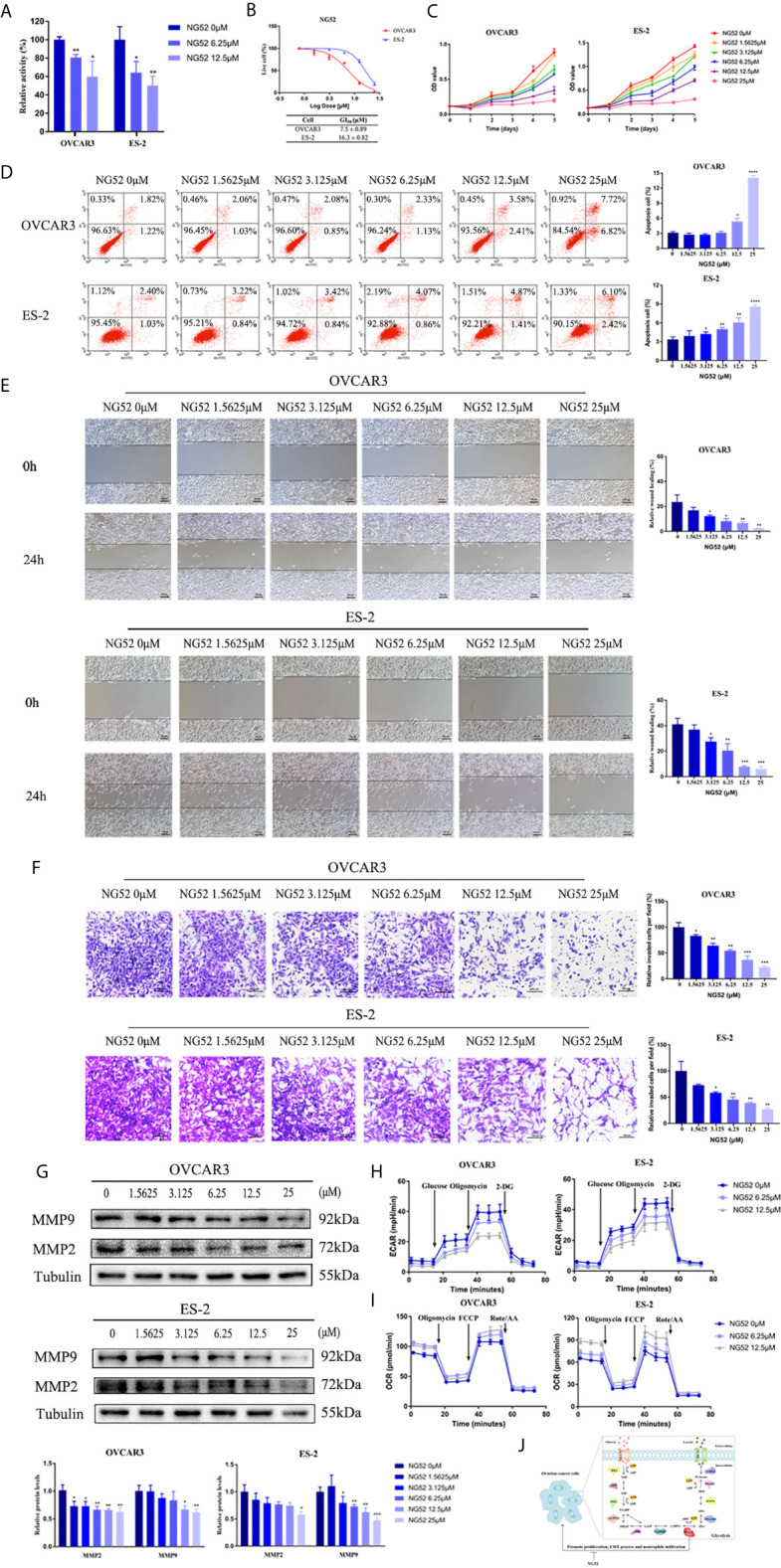
The effect of NG52 on ovarian cancer cells. **(A)** The effect of NG52 on PGK1 activity. **(B)** NG52 inhibited the proliferation of ovarian cancer cells in a dose-dependent manner. **(C)** Time points of NG52 anti-proliferative effects on ovarian cancer cells. **(D)** NG52 induced apoptosis of ovarian cancer cells. **(E)** NG52 reduced the migration of ovarian cancer cells. **(F)** NG52 reduced the invasion of ovarian cancer cells. **(G)** NG52 decreased the protein expression levels of MMP2 and MMP9. **(H)** Chart of ECAR measurement in cells treated with NG52. **(I)** Chart of OCR measurement in cells treated with NG52. **(J)** Graphical abstract. Error bars represent the mean ± SD of three independent experiments. *P < 0.05; **P < 0.01; ***P < 0.001, ****P < 0.0001.

Recently, NG52, a yeast cell cycle regulating kinase inhibitor, has been identified as a PGK1 inhibitor. Results showed that NG52 attenuated the kinase activity of PGK1 in ovarian cancer cells (**Figure 9A**). Due to the high PGK1 expression in ovarian cancer, the anti-proliferative effect of NG52 in various ovarian cancer cell lines was investigated. Results showed that NG52 could inhibit the proliferation of OVCAR3 and ES-2 cell lines in a dose-dependent manner (**Figure 9B**). When cells were cultured with different NG52 concentrations, the proliferation ability was inhibited on the second day after treatment (**Figure 9C**). We next evaluated the effect of NG52 on apoptosis. Flow cytometry results revealed that the overall apoptosis of OVCAR3 and ES-2 cell lines was significantly increased after treated with NG52 for 3 days (**Figure 9D**). Furthermore, NG52 significantly reduced the migration and invasion ability of OVCAR3 and ES-2 cell lines (**Figures 9E, F**). Western blot analysis indicated that the expression levels of MMP2 and MMP9 decreased after treated with NG52 (**Figure 9G**). To further confirm the effect of NG52 on cell metabolic process, ECAR and OCR were evaluated. Results showed that NG52 suppressed the glycolytic capability of OVCAR3 and ES-2 cells in a concentration dependent manner (**Figure 9H**). On the contrary, the OCR parameters increased after NG52 administration (**Figure 9I**). These results suggested that PGK1 would become a powerful therapeutic target for anti-glycolysis therapy in ovarian cancer (**Figure 9J**).

## Discussion

Metabolic reprogramming is currently a research hotspot in the field of oncology. Cancer cells will respond to a variety of exogenous and endogenous signals to obtain metabolic adaptability. By adjusting metabolism and nutrition, tumor cells maintain a stable redox environment, rapid ATP synthesis, and rapid biomacromolecule synthesis, thereby maintaining their continuous proliferation (20). Glycolysis is a classic example of metabolic reprogramming. Even under conditions of sufficient oxygen, tumor cells can take in a large amount of glucose for glycolysis (8). Therefore, understanding how glycolysis promotes tumor progression and which activity plays the most critical role in glycolysis can provide new research ideas for targeted therapy of cancer. In the present study, database analyses and immunohistochemical assays were combined to evaluate the expression levels and prognostic value of metabolite transporters and enzymes involved in the glycolytic process of ovarian cancer. Furthermore, the mechanism of PGK1 and the effect of NG52 were investigated. We hypothesize that PGK1 will become a powerful target for anti-glycolysis therapy in ovarian cancer.

Selective inhibition of glycolysis can inhibit the progression of malignant tumors by depriving energy requirements. Currently, research targets mainly focus on metabolite transporters and glycolytic rate-limiting enzymes. Preclinical and early clinical studies have confirmed the effectiveness of this treatment (9). However, anti-glycolysis therapy has not yet been transformed into clinical applications, and the specific targets in ovarian cancer need to be further clarified. Therefore, Oncomine and GEPIA databases were used to comprehensively analyze the expression levels of glycolysis-related genes in ovarian cancer. The results showed that most glycolysis-related genes, including *SLC2A1*, *SLC16A3*, *HK2*, *GPI*, *PFKP*, *TPI1*, *GAPDH*, *PGK1*, *ENO1*, *PKM2*, and *LDHA*, were highly expressed in ovarian cancer. In addition, the HPA database was used to verify the protein expression levels in ovarian cancer. Consequently, different histological types of ovarian cancer and normal ovarian tissue specimens were obtained from the hospital to compare the expression level of the above-mentioned high-expression indicators. The experimental results were consistent with the database analysis results. In addition, GLUT1, HK2, PFKP, PGK1, and PKM2 had higher expression levels in mucinous carcinoma. GLUT1, MCT4, PFKP, PGK1, and PKM2 had lower expression levels in endometrioid carcinoma. Therefore, different glycolysis indicators can be used as therapeutic targets for various histological types of epithelial ovarian cancer. However, this result may be affected by individual differences and needs further validation using a large study cohort.

The increase in metabolic activity is closely associated with tumor aggressiveness, and higher activated glycolysis indicates a poor prognosis for tumor patients. Patient prognosis was assessed in numerous studies by establishing prognostic risk signatures that combined multiple metabolism-related genes (21, 22). Targeting tumor metabolism was proposed to hopefully improve patient prognosis. High HK2 expression in ovarian cancer was an independent predictor of disease-free survival (DFS) as confirmed in the literature. In addition, high PKM2 expression was closely associated with poor PFS, and correlated with short OS in platinum-treated epithelial ovarian cancer (23–25). For metabolite transporters, the expression level of GLUTs was not a prognostic factor of ovarian cancer, however, MCT4 negatively correlated with survival of mice confirmed based on *in situ* metabolic profiling of ovarian cancer tumor xenografts (26, 27). However, the prognostic value of other indicators in ovarian cancer has not been extensively explored. Therefore, the correlation between the above-mentioned glycolysis-related genes and prognosis of patients with ovarian cancer was evaluated to identify the therapeutic target. Kaplan-Meier plotter analysis indicated that among the highly expressed glycolysis genes, *SLC16A3*, *HK2*, *GPI*, *PFKP*, and *PGK1*, were closely associated with poor PFS, PPS, or OS in patients with ovarian cancer. *SLC16A3*, *PFKP*, and *PGK1*, which were highly expressed and associated with poor prognosis, were further screened to evaluate the correlation between their expression levels and the prognosis of patients with different stages and differentiation. *PGK1* had the greatest prognostic value and showed an even more significant correlation with prognosis in patients with moderate differentiation and advanced FIGO stages. Therefore, among glycolysis-related genes, *PGK1* may play the most critical role in the glycolysis process of ovarian cancer, and targeted drugs for *PGK1* may effectively improve the survival of patients with ovarian cancer.

PGK1, the first ATP-producing enzyme in the glycolysis process, can catalyze the conversion of 1,3-diphosphoglycerate and ADP into 3-phosphoglycerate (3-PG) and ATP, thereby playing an important role in biosynthesis and redox balance (10). In addition, post-translational modifications of PGK1, such as phosphorylation and glycosylation, induce the translocation of PGK1 into mitochondria, leading to pyruvate dehydrogenase kinase 1 (PDHK1) Thr338 phosphorylation and suppression of mitochondrial oxidative phosphorylation. This results in an increase in extracellular acidification and lactate production, thereby promoting tumorigenesis (28, 29). PKM2 is another ATP-producing enzyme. Notably, cancer cells were shown in previous studies to likely prefer the less active PKM2, leading to the accumulation of upstream glycolytic metabolites, thereby meeting requirements of nucleic acids, amino acids, and lipids (30). Therefore, PGK1 plays a key role in the ATP synthesis of cancer cells and the promotion of tumor progression. PGK1 overexpression was confirmed to be associated with multidrug resistance in ovarian cancer (31). In addition, ACTL6A participated in follicle-stimulating hormone (FSH)-driven glycolysis in ovarian cancer cells by upregulating PGK1 (32). However, the prognostic value and mechanism of PGK1 in ovarian cancer remain unclear. The present study included a large number of clinical specimens to confirm the expression level and prognostic value of PGK1 in ovarian cancer. Immunochemistry assays showed that PGK1 was highly expressed in epithelial ovarian cancer, especially in mucinous carcinoma. In addition, PGK1 expression level was correlated with the degree of differentiation. COX regression model analysis indicated that high PGK1 expression was an independent risk factor affecting the survival and prognosis in patients with epithelial ovarian cancer. Further biological functional experiments confirmed that inhibition of PGK1 expression in ovarian cancer cells can reduce the EMT process, resulting in loss of cell migration and invasion ability. Therefore, PGK1 has the greatest correlation with prognosis in patients with ovarian cancer and is closely associated with tumorigenesis and progression.

The aerobic glycolysis and resultant acidification of the tumor microenvironment will affect the anti-tumor immune response mediated by T cells, promote immune escape, and attract tumor-promoting inflammatory cells (33). Data analysis showed that in addition to metabolic reprogramming pathways, neutrophil activation and chemokine signaling pathways were closely associated with *PGK1*. Studies showed that neutrophils promote tumor progression by inhibiting T cell activation and promoting genetic mutation, tumor cell proliferation, angiogenesis, and metastasis (34). The latest research confirms that neutrophils interact with circulating tumor cells to support cell cycle progression and accelerate metastatic seeding (35). At present, studies have confirmed the correlation between PGK1 and immune/inflammation cells in breast and lung cancer. However, its relationship with neutrophils needs further evaluation (36, 37). Consequently, TISIDB and TIMER databases were used to evaluate the correlation of *PGK1* with immune and inflammatory cell infiltration. Results showed that neutrophils had the strongest correlation with *PGK1*. In addition, among the several infiltrating cells with the strongest correlation with *PGK1*, neutrophils, dendritic cells, monocytes, and macrophages may serve as inflammatory cells that contribute to the progression of malignancies and are associated with poor prognosis (38). Among the several chemokines with the strongest correlation with *PGK1*, CXCL8 and CCL2 are major metastasis-promoting inflammatory chemokines. High expression of CXCL8 enhanced neutrophils recruitment, thereby promoting EMT process and immune evasion in multiple cancers (39, 40). Therefore, *PGK1* may affect the recruitment of neutrophils by regulating the level of CXCL8, leading to the progression of ovarian cancer. However, further experiments are needed to validate the significance of *PGK1* in regulating neutrophil activation and recruitment.

Currently, several potential PGK1 inhibitors are under development. Some inhibitors can indirectly inhibit the enzyme activity or protein expression level of PGK1. For example, acetyltransferase inhibitors or sirtuin activators inhibit PGK1 enzyme activity by regulating the post-translational modification of PGK1 (41). Bisphosphonate analogues act as analogues of 1,3-BPG to reduce the enzymatic activity of PGK1 (42). Treatment with the newly synthesized small-molecule compound, CBR-470-1, increases metabolite levels upstream of PGK1 (43). However, low activity and poor specificity, as well as possible toxic and side effects on normal cells, limit their clinical application. The inhibitory effect of several inhibitors on tumors needs further evaluation. NG52 is a yeast cell cycle-regulating kinase inhibitor. In recent studies, NG52 was shown to target the kinase activity of PGK1 and reverse the Warburg effect by inhibiting the phosphorylation of PDHK1 at residue Thr338 site and enhancing the activity of pyruvate dehydrogenase (PDH) in glioma cells (44). Our research data showed that NG52 could exhibit the inhibitory effect on PGK1 kinase activity to impede Warburg effect. In addition, NG52 can inhibit the growth of ovarian cancer cells and induce apoptosis in a dose-dependent manner. The results showed that OVCAR3 cells were more sensitive to NG52 than ES-2 cells. When treated with high concentration of NG52, the apoptotic ratio of OVCAR3 cells was higher than that of ES-2 cells. An increasing number of studies have confirmed the interaction between the Warburg effect and EMT process. A favorable reprogramming of metabolism can provide a survival advantage for metastatic cancer cells, while EMT inducers also affect various metabolic processes (45). Our research data showed that NG52 could reverse the EMT phenotype and regulate the expression of EMT-related proteins in ovarian cancer cells. NG52 decreased the level of ECAR and increased the level of OCR, which manifested that glycolysis was dampened. Database analysis suggested that *PGK1* was correlated with oxidoreductase activity. Studies showed that metabolic reprogramming is coupled with redox perturbation in cancers (46). As one of the products of PGK-catalyzed reaction, 3-PG can be oxidized and thus enter one-carbon metabolism, ultimately affecting the redox balance (47). In addition, increasing PDH activity suppresses cancer cell proliferation by reducing the NAD+/NADH ratio (48). Therefore, we speculate that NG52 may affect PDH activity by regulating its phosphorylated state, thereby regulating the redox balance.

In summary, bioinformatics analysis and a large number of clinical specimens were combined in the present study to confirm the expression levels of glycolysis-related metabolite transporters and enzymes in ovarian cancer. PGK1, which showed the best prognosis value, was selected for further analysis. The function of PGK1 in ovarian cancer was clarified and the value of NG52 as a targeted drug was proposed. Therefore, PGK1 can be considered as an attractive molecular target for anti-glycolytic therapy of ovarian cancer.

## Data Availability Statement

The original contributions presented in the study are included in the article/**Supplementary Material**. Further inquiries can be directed to the corresponding author.

## Ethics Statement

Written informed consent was obtained from the individual(s) for the publication of any potentially identifiable images or data included in this article.

## Author Contributions

RG and BL conceived and designed the study. RG performed the experiments and wrote the manuscript. YH, OL, and HD contributed to the sample collection. LG, SW, MZ, and XL contributed to the data collection. BL revised the manuscript. All authors contributed to the article and approved the submitted version.

## Funding

This work was supported by the Shengjing Free Researchers’ Plan (201804) and the Key R&D Guidance Plan Project in Liaoning Province (2019JH8/10300022).

## Conflict of Interest

The authors declare that the research was conducted in the absence of any commercial or financial relationships that could be construed as a potential conflict of interest.
