# Supplementary material for: PGK1 Is a Key Target for Anti-Glycolytic Therapy of Ovarian Cancer: Based on the Comprehensive Analysis of Glycolysis-Related Genes
Source: Front Oncol. 2021 Jul 1;11:682461. doi: 10.3389/fonc.2021.682461 (PMC8281930; doi:10.3389/fonc.2021.682461)
Supplement: Supplementary file 1 [file DataSheet_1.pdf]

Additional file 1. Antibodies used in the immunochemistry assay

Antibody	Concentration	Catalogue NO.	Company
GLUT1	1:100	21829-1-AP	Proteintech (Wuhan, China)
MCT4	1:50	22787-1-AP	Proteintech (Wuhan, China)
HK2	1:400	22029-1-AP	Proteintech (Wuhan, China)
GPI	1:300	15171-1-AP	Proteintech (Wuhan, China)
PFKP	1:600	13389-1-AP	Proteintech (Wuhan, China)
TPI1	1:600	10713-1-AP	Proteintech (Wuhan, China)
GAPDH	1:1500	10494-1-AP	Proteintech (Wuhan, China)
PGK1	1:250	17811-1-AP	Proteintech (Wuhan, China)
ENO1	1:600	11204-1-AP	Proteintech (Wuhan, China)
PKM2	1:300	15822-1-AP	Proteintech (Wuhan, China)
LDHA	1:400	21799-1-AP	Proteintech (Wuhan, China)
